# Topographic separation of fornical fibers associated with the anterior and posterior hippocampus in the human brain: An MRI‐diffusion study

**DOI:** 10.1002/brb3.604

**Published:** 2016-11-22

**Authors:** Kat Christiansen, Claudia Metzler‐Baddeley, Greg D. Parker, Nils Muhlert, Derek K. Jones, John P. Aggleton, Seralynne D. Vann

**Affiliations:** ^1^ School of PsychologyCardiff UniversityCardiffUK; ^2^ Cardiff University Brain Research Imaging Centre (CUBRIC)Neuroscience and Mental Health Research Institute (NMHRI)Cardiff UniversityCardiffUK; ^3^ School of Psychological SciencesUniversity of ManchesterManchesterUK

**Keywords:** amnesia, fornix, memory, Papez circuit, tractography

## Abstract

**Background and Objective:**

Evidence from rat and nonhuman primate studies indicates that axons comprising the fornix have a characteristic topographical organization: projections from the temporal/anterior hippocampus mainly occupy the lateral fornix, whereas the more medial fornix contains fibers from the septal/posterior hippocampus. The aim of this study was to investigate whether the same topographical organization exists in the human brain.

**Methods:**

Using high angular resolution diffusion MRI‐based tractography at 3T, subdivisions of the fornix were reconstructed in 40 healthy adults by selecting fiber pathways from either the anterior or the posterior hippocampus.

**Results:**

The tract reconstructions revealed that anterior hippocampal fibers predominantly comprise the lateral body of the fornix, whereas posterior fibers make up the medial body of the fornix. Quantitative analyses support this medial:lateral distinction in humans, which matches the topographical organization of the fornix in other primates.

**Conclusion:**

This novel tractography protocol enables the separation of fornix fibers from anterior and posterior hippocampal regions in the human brain and, hence, provides a means by which to compare functions associated with different sets of connections along the longitudinal axis of the hippocampus.

## 1 Introduction

The fornix is the principal white matter tract connecting the hippocampal formation with areas beyond the temporal lobe, including prefrontal cortex, the anterior thalamic nuclei, the mammillary bodies, the ventral striatum, and the basal forebrain (Aggleton, 2008; Poletti & Creswell, 1977). Neuropsychological investigations of patients with fornix damage first highlighted the importance of this tract for episodic memory (Aggleton et al., 2000; D'Esposito, Verfaellie, Alexander, & Katz, 1995; Gaffan & Gaffan, 1991; Vann et al., 2008). Neuroimaging findings from healthy participants, as well as from patients with fornix pathology, have further established the importance of this tract for episodic memory (Metzler‐Baddeley, Jones, Belaroussi, Aggleton, & O'Sullivan, 2011; Metzler‐Baddeley, Hunt, et al., 2012; Oishi, Mielke, Albert, Lyketsos, & Mori, 2012; Zhuang, Sachdev, et al., 2012; Zhuang, Wen, et al., 2012). Despite the role of this tract in cognition, surprisingly little is understood about the organization of the fibers within the human fornix and how this might relate to its various connections. The principal motivation to examine their topography arises from the growing evidence for functional differences along the anterior–posterior axis of the hippocampus (Collin, Milivojevic, & Doeller, 2015; Fanselow & Dong, 2010; Poppenk, Evensmoen, Moscovitch, & Nadel, 2013; Strange, Witter, Lein, & Moser, 2014). For instance, evidence from functional neuroimaging studies suggests a role of the posterior hippocampus in spatial navigation (e.g., Hartley, Maguire, Spiers, & Burgess, 2003), whereas anterior hippocampus has been associated with goal‐directed spatial decision making (Viard, Doeller, Hartley, Bird, & Burgess, 2011). Thus, a method that would allow the anatomical dissociation of fibers associated with anterior and posterior hippocampal regions in humans would aid the study of functional dissociation between these different hippocampal networks.

In macaque monkeys, the projections from the anterior hippocampus mainly occupy the lateral fornix, whereas the more medial fornix contains fibers from the posterior hippocampus (Saunders & Aggleton, 2007). A similar organization exists in the rat, whereby fibers from the temporal hippocampus (equivalent to the primate anterior hippocampus) are located more laterally within the fornix, whereas fibers from the more septal hippocampus (equivalent to the posterior hippocampus) are found more medially (Swanson & Cowan, 1977; Wyss, Swanson, & Cowan, 1980). It is not yet known if the human fornix has a similar topography, even though such information could provide a useful means to compare the respective functions of the anterior and posterior hippocampal networks (Aggleton, 2012; Strange et al., 2014).

This study, therefore, employed the damped Richardson‐Lucy algorithm (Dell'acqua et al., 2010) for deterministic tractography on high angular resolution diffusion imaging data (HARDI) (Tuch et al., 2002) to visualize those axons linked, respectively, to the anterior hippocampus and to the posterior hippocampus. The extent of overlap between the reconstructions was then determined quantitatively. In addition, various white matter microstructural properties of these two subpopulations of fornical fibers were characterized by diffusion tensor‐based indices of fractional anisotropy (FA), radial diffusivity (RD) (Basser, Mattiello, & LeBihan, 1994; Pierpaoli & Basser, 1996), tissue volume fraction (*f*) (Pasternak, Sochen, Gur, Intrator, & Assaf, 2009), and by the hindrance‐modulated orientational anisotropy (HMOA) (Dell'Acqua, Simmons, Williams, & Catani, 2013). The HMOA provides a novel fiber population‐specific index of the diffusion properties along the reconstructed pathways, which may be more sensitive to inter‐individual differences in white matter microstructure than tensor‐based metrics (Christiansen, Aggleton, et al., 2016; Dell'Acqua et al., 2013). The purpose of comparing these various indices between the two populations of fornical fibers (anterior and posterior hippocampus) was to appreciate if these potentially distinct pathways might be distinguishable in ways additional to their physical location.

## 2 Materials and Methods

### 2.1 Participants

Forty healthy participants were recruited from Cardiff University volunteer databases and from the local community via poster advertisements. Participants were between 19 and 40 years of age (mean age = 26.60; standard deviation = 6.46; 21 women, 1 left‐handed) without any known history of neurological or psychiatric illness, head injury, drug/alcohol abuse, or MRI contraindications as obtained by self‐report. All participants underwent cognitive assessment in the Cambridge Brain Sciences Laboratory tasks (Hampshire, Highfield, Parkin, & Owen, 2012) and performed within normal ranges for their age group (please see Table 1 in Metzler‐Baddeley, Caeyenberghs, Foley, & Jones, 2016).

### 2.2 Diffusion‐weighted MRI and T_1_‐weighted MRI scanning protocols

The MRI data were acquired at the Cardiff University Brain Research Imaging Centre (CUBRIC) with a 3T General Electric HDx MRI system (GE Medical Systems, Milwaukee) using an eight channel receiver only head RF coil. The MRI protocol consisted of the following imaging sequence: A high‐resolution T_1_‐weighted anatomical scan (FSPGR) (256 × 256 acquisition matrix, TR = 7.8 ms, TE = 2.9 ms, flip angle = 20, 172 slices, 1 mm slice thickness, FOV = 23 cm). Diffusion data were acquired employing a spin‐echo echo‐planar HARDI sequence with diffusion encoded along 60 isotropically distributed orientations and 6 nondiffusion‐weighted scans according to an optimized gradient vector scheme (Jones, Horsfield, & Simmons, 1999) (Field of view 230 × 230 mm, 96 × 96 acquisition matrix, TR/TE = 87 ms, b‐value = 1200 s/mm^2^, 60 slices, 2.4 mm slice thickness, reconstructed spatial resolution 1.8 × 1.8 × 2.4 mm). Data acquisition was peripherally gated to the cardiac cycle with a total acquisition time of ~30 min depending on the heart rate.

The diffusion‐weighted HARDI data were corrected for distortions induced by the diffusion‐weighted gradients, artifacts due to head motion, and due to EPI‐induced geometrical distortions by nonlinearly registering each image volume to their T_1_‐weighted anatomical images (resulting in a reconstructed spatial resolution of 1 × 1 × 1 mm) (Irfanoglu, Walker, Sarlls, Marenco, & Pierpaoli, 2012), with appropriate reorientation of the encoding vectors (Leemans & Jones, 2009) in ExploreDTI (Version 4.8.3) (Leemans, Jeurissen, Sijbers, & Jones, 2009). A two compartment model using the Free Water Elimination (FWE) approach (Pasternak et al., 2009) to correct for any partial volume artifacts in the diffusion metrics (Metzler‐Baddeley, O'Sullivan, Bells, Pasternak, & Jones, 2012) was fitted to derive maps of FA, RD and *f* (Metzler‐Baddeley, O'Sullivan, et al., 2012; Pasternak et al., 2009).

### 2.3 Tractography and tract‐specific measures

Whole‐brain tractography was performed for each participant using the damped Richardson‐Lucy spherical deconvolution algorithm (Dell'acqua et al., 2010), which allows the recovery of multiple fiber orientations within each voxel, including those affected by partial volume. The tracking algorithm estimated peaks in the fiber orientation density function (fODF) at the center of each image voxel, and seed points were positioned at the vertices of a 2 × 2 × 2 mm grid superimposed over the image. Streamlines along the orientation of the fODF peaks were then generated in 0.5 mm steps and fODF peaks were reestimated at each new location (Jeurissen, Leemans, Jones, Tournier, & Sijbers, 2011). Tracts were terminated if the fODF threshold fell below 0.05 or the direction of pathways changed through an angle greater than 45° between successive 0.5 mm steps. This procedure was then repeated by tracking in the opposite direction from the initial seed point. Streamlines outside a minimum of 10 mm and maximum of 500 mm length were discarded. At each 0.5 mm step, local estimates of FA, RD, and *f* were acquired through interpolation of associated parameter maps while HMOA was captured at the time of streamline generation by recording the minimally subtending local fODF peak magnitude with appropriate normalization (Dell'Acqua et al., 2013).

### 2.4 Reconstruction of anterior/posterior hippocampal fornices

Reconstructions of the fornix were based on an anterior/posterior split of the hippocampus using each participant's T_1_‐weighted scan. Region of interests (ROIs) were defined following Boolean logic by placing “SEED” (either/or), “AND” (inclusive), or “NOT” (exclusive) waypoint gates.

#### 2.4.1 Anterior hippocampal fornix

**Figure 1:**
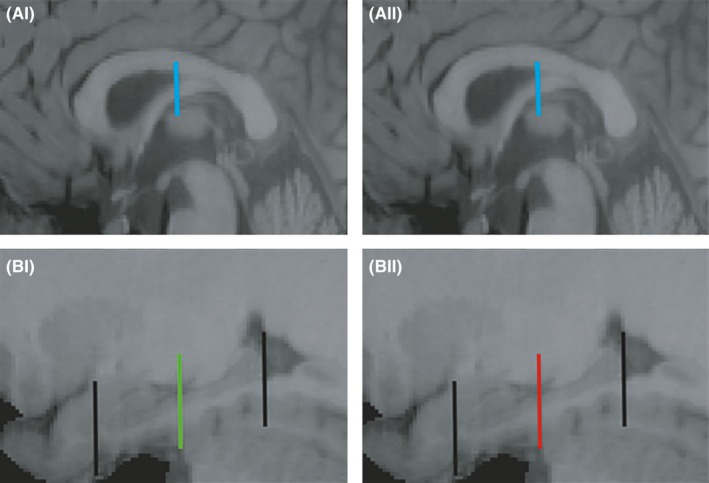
(A, B) Parasagittal images showing the shared landmarks used for reconstructing the anterior (A‐I, B‐I) and posterior (A‐II, B‐II) hippocampal fornix. The same SEED gate is first placed around body of fornix (sagittal plane, blue bar A‐I, II). Midway between the head of the uncus and the tail of the hippocampus (both marked with a black bar), a bar was placed as either an AND gate (B‐I, green, anterior hippocampus) or a NOT gate (B‐II, red, posterior hippocampus), using the lateral‐most sagittal plane point where the uncus is still visible

For the segment of the fornix associated with the anterior portion of the hippocampus, a “SEED” ROI was placed around the body of the fornix on the coronal plane 6 mm posterior to the anterior commissure, as defined by that participant's T_1_‐weighted scan (Figure 1A‐I, blue bar). An “AND” ROI was placed halfway along the length of the left or right hippocampus, respectively, on the coronal plane as shown in Figure 1B‐I (green bar). Consequently, the tract reconstructions reflected those fiber pathways that reach or extend anterior to this “AND” ROI. For consistency, the same coronal section provided the “AND” ROI for the two hemispheres. The microstructural indices (FA, RD, *f*, and HMOA) were averaged along the tract reconstructions that were jointly compiled from both hemispheres, so giving a single overall mean value for each participant for each measure.

#### 2.4.2 Posterior hippocampal fornix

For the posterior hippocampal fornix, the procedure was identical to that described above except that a “NOT” gate, instead of an “AND” ROI, was placed at the mid‐hippocampal level (Figure 1B**‐**I, red bar). This NOT gate should exclude those fibers continuing anteriorly beyond this point, that is, only fibers associated with the posterior half of the hippocampus should be reconstructed. The microstructural indices were calculated as for the anterior hippocampal fornix.

For both the anterior and posterior hippocampal fornix fiber reconstructions, “NOT” ROIs were applied to exclude any extraneous fibers not consistent with the known fornix anatomy. These NOT ROIs were placed as follows using the midline sagittal plane for reference: i. on the coronal slices immediately anterior to the genu of the corpus callosum and immediately posterior to the splenium of the corpus callosum, ii. on the axial slices at the level of the lower limit of the body of the corpus callosum and at the level of the upper limit of the pons, and iii. on the sagittal slices lateral to the fornix at the edge of the medial temporal lobe for each hemisphere.

**Figure 2:**
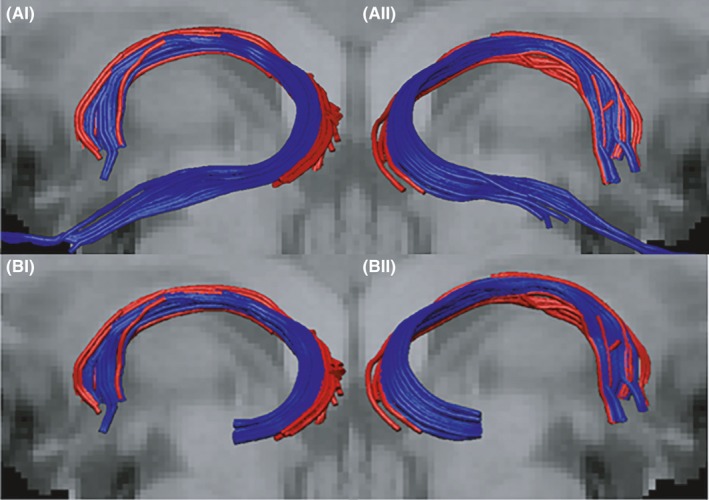
(A) Examples of fornix fiber streams for the anterior (blue) and posterior (red) hippocampus from a left sagittal (I) and right sagittal (II) view. (B) Examples of the “truncated” anterior fornix fiber streams (blue) accompanied by the full posterior hippocampal streams (red). (Anterior hippocampal fornices were cut at the AND/NOT gate levels depicted in Figure 1A‐II, B‐II)

Before calculating and comparing the four microstructural indices for the anterior and posterior hippocampal reconstructions, the anterior hippocampal fornix streams were truncated at the posterior hippocampus, so that they began at the same coronal level as the posterior streams (Figure 2B‐I,II). This procedure excluded the additional part of the anterior hippocampal fornix (along the length of the hippocampus) that would otherwise make the comparison nonequivalent. In practice, fibers relating to the anterior hippocampal reconstructions were cut short along the body of the hippocampus, at the level of the AND gate, using the “splitter tool” function in ExploreDTI.

To provide an initial visualization of the reconstructed anterior and posterior hippocampal fornix tracts for all participants, all reconstructions were converted into Nifti format and warped into the Montreal Neurological Institute (MNI) standard 2‐mm FA template for diffusion‐weighted scans. All Nifti images were combined into one composite image across participants for each tract and the mean of this composite image was computed using FSL FMRIB software.

### 2.5 Statistical analysis

All statistical analyses were carried out using SPSS v. 20 (IBM Corp, 2011). All micro‐ and macrostructural data for each tract were inspected for outliers, defined as values larger than three times the absolute z‐score from the mean.

### 2.6 Assessment of overlap between fornix subdivisions

**Figure 3:**
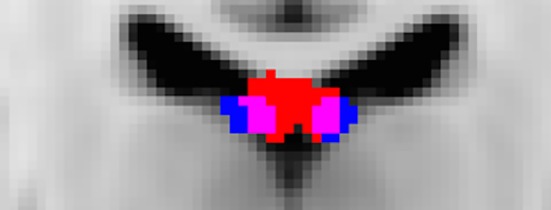
Mean MNI normalized voxels across all participants for the anterior hippocampal fornix reconstruction (blue) and the posterior hippocampal fornix reconstruction (red). Voxels containing both tracts are shown in pink. (Coronal section: X = 89 on MNI 1 mm T1‐weighted FMRIB template)

In addition to visualizing the MNI‐transformed results for the anterior and posterior hippocampal reconstructions (Figure 3), two voting algorithms were applied to the data. For both methods, the participants’ T_1_‐weighted images were first coregistered nonlinearly with Elastix (Klein, Staring, Murphy, Viergever, & Pluim, 2010) to the MNI 1 × 1 × 1 mm template and then transformed using individual participant's anterior hippocampal and posterior hippocampal fornix tract masks (3D volumes in which voxels intersected by a streamline are set to 1, all others to 0) into a common space using the same warp fields. The overlap between the two fornix reconstructions was then investigated using the following two methods.

The first method (‘probability based’) assigned a varying shade between red and blue to each voxel occupied by the anterior or posterior hippocampal fornix (blue for anterior and red for posterior). The shade was determined by the ratio of participants who possessed either tract within this voxel, for example, if more participants possessed the anterior hippocampal fornix in a given voxel than posterior hippocampal fornix, then this voxel would be a bluer shade rather than a redder shade. In the case of a 50/50 split, the voxel color was halfway between red and blue, that is, purple.

The second illustrative method (‘winner takes all’) used the same initial process, but then applied a ‘winner takes all’ binary voting scheme to the probability‐based data. Consequently, voxels were assigned either to the anterior hippocampal or the posterior hippocampal fornix according to whether they received more projections from the anterior or posterior hippocampus across participants. Voxels with equal numbers of projections from anterior and posterior hippocampus are illustrated in purple.

Quantitative measures of overlap between the anterior and posterior hippocampal‐derived fornices were also calculated (Dice, 1945). A Dice score was calculated for each individual using the following formula (where *x* is the Dice score, *A* and *B* are two separate tract masks, and *C* is the overlap between them): *x *=* *2*C*/(*A*+*B*). Dice scores vary between 0 and 1, the higher the score the greater the level of overlap between the samples.

### 2.7 Relationship of microstructural indices from the two fornix subdivisions

When comparing the four microstructural indices for the anterior and posterior hippocampal reconstructions, the anterior hippocampal data came from truncated reconstructions (see above) so that they began at the same coronal level as the posterior streams (Figure 2B‐I, B‐II).

The four microstructural indices (FA, HMOA, *f,* RD) for the anterior and the posterior hippocampal fornix subdivisions were separately compared using paired sample *t*‐tests (two‐tailed) and correlational analyses (Pearson's r correlations). These comparisons were always restricted within the same index, for example, making four *t*‐tests in total. Consequently, a Bonferroni adjusted alpha level of 0.0125 (i.e., 0.05 alpha level divided by four comparisons) helped to determine significance.

## 3 Results

### 3.1 Anterior and posterior hippocampal fornix reconstructions

**Figure 4:**
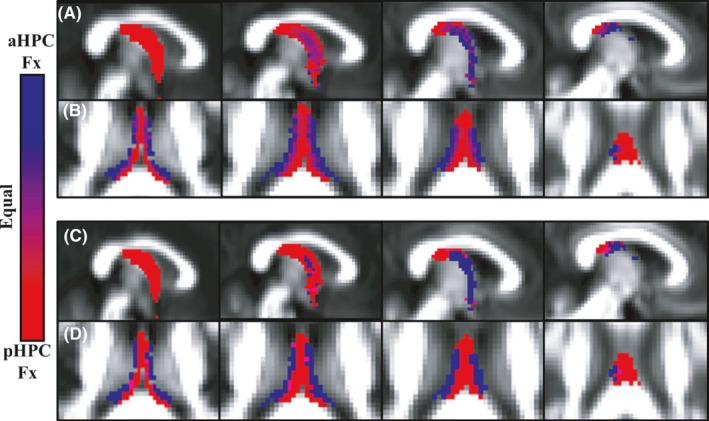
(A, B) Relative probability maps depicting voxels that “belong” to the anterior (aHPCFx, blue) or posterior (pHPCFx, red) hippocampal fornix, with purple as equal ownership. The images in (A) go from the midline (left) to the lateral (right) parasagittal plane (MNI 2 mm *y*‐axis slices 45–48). The images in (B) proceed vertically from MNI z‐axis slice 44–47, that is, inferior (left) to superior (right). (C, D) Binary segmentations of anterior and posterior hippocampal fiber streams according to a winner‐takes‐all scheme (blue = anterior; red = posterior). Other conventions are the same as in (A and B)

Both fornix subdivisions could be reconstructed in all 40 participants for both hemispheres. Based on the outlier criterion (see Methods), one value for the anterior hippocampal fornix RD (n = 39) and two values for the posterior hippocampal fornix *f* (n = 38) were excluded from the analyses. For all remaining participants, the anterior hippocampal fiber streams occupied a lateral position within the body of the fornix, whereas the posterior hippocampal fiber streams were located in the medial portion of the fornix (Figures 3 and 4).

### 3.2 Visual representation: Anterior versus posterior hippocampal fornix

The outcome of the ‘probability based’ model is shown in Figure 4A,B. The areas of overlap between the tracts are illustrated by the purple hues. It is evident that the two structures remain largely separable, particularly in the z‐axis. The results of the ‘winner take all’ method (Figure 4C,D) binarize the same voxels according to the voting scheme described above. This binarized image demonstrates how few voxels contribute equally to either tract.

### 3.3 Dice scores and overlap

The median Dice score for the anterior and posterior hippocampal fornix was 0.23 (lower quartile, 0.16; upper quartile, 0.30). Likewise, the mean Dice score was similarly low (0.23; S.D. 0.09), again reflecting the low voxel overlap between the subdivisions (Cabezas, Oliver, Llado, Freixenet, & Cuadra, 2011; Van Leemput et al., 2009).

### 3.4 Correlations and t‐test comparisons between diffusion measures

Significant positive correlations (all *p *< .0125 Bonferroni corrected) were found between the anterior and posterior hippocampal fornices for each of the four microstructural measures (Pearson r: FA 0.62, RD 0.75, *f* 0.68, HMOA, 0.64). Despite these correlations, within‐subject *t*‐tests revealed differences in these same measures (all *p *< .001) between the anterior (truncated) hippocampal fornix [mean and standard deviation: FA 0.37 (0.03), RD 0.86 (0.06) *x10* ^*3*^, *f* 0.70 (0.03), HMOA 0.25 (0.03)], and the posterior hippocampus [FA 0.39 (0.02), RD 0.91 (0.11) *x10* ^*3*^, *f* 0.66 (0.03), HMOA 0.27 (0.04)].

## 4 Discussion

The fornix is the principal white matter tract associated with the hippocampal formation. However, despite its status, relatively little is known about the organization of fibers within this tract in humans. In both nonhuman primates and rats, a topography exists along the medial–lateral axis of the fornix that relates to the longitudinal axis of the hippocampus. To determine whether a similar topography exists in the human fornix, we used deterministic tractography to reconstruct fornix fibers associated with either the anterior or posterior hippocampus. A clear distinction was found as fibers associated with the anterior hippocampus were located laterally within the body of the fornix, whereas fibers associated with the posterior hippocampus were found medially. Thus, we found the same topographical organization as has been reported previously in nonhuman primates and rats (Meibach & Siegel, 1977; Saunders & Aggleton, 2007). Formal analyses of the two fiber populations showed little overlap between the pathways associated with the anterior and posterior hippocampus.

Current diffusion‐based MRI methods are unable to determine the direction of any particular connection, that is, they cannot distinguish hippocampal efferents from afferents within the fornix. Nevertheless, it is most likely that the MRI signals principally reflect the topography of hippocampal efferents as the reciprocal afferent fibers are far less numerous within the tract (Saunders & Aggleton, 2007). These hippocampal efferents principally arise from the CA fields and the subicular cortices (Chase et al., 2015; Saunders & Aggleton, 2007). For this reason it can be inferred that the more lateral fornix fibers preferentially innervate targets like the prefrontal cortex, nucleus accumbens, and the anteromedial thalamic nucleus, as in every instance the anterior hippocampus provides the most numerous inputs to these sites (Aggleton, Wright, Rosene, & Saunders, 2015; Barbas & Blatt, 1995; Chase et al., 2015; Christiansen, Dillingham, et al., 2016). In contrast, more posterior hippocampal projections in the fornix include the dense inputs to the mammillary bodies (Christiansen, Dillingham, et al., 2016). It should, however, be emphasized that in the rat and macaque brains these distinctions are relative, that is, there is a gradient in the anterior–posterior inputs from the subiculum and CA1 rather than a sharp division between the anterior and posterior hippocampus (Aggleton, 2012; Barbas & Blatt, 1995; Evensmoen et al., 2015; Kjelstrup et al., 2008; for review see Strange et al., 2014).

Studies with monkeys have also shown that some hippocampal afferents are organized topographically within the fornix (Saunders & Aggleton, 2007). Injections of a retrograde tracer directly into the fornix demonstrated how the basal forebrain projections to the hippocampus have a medial–lateral organization within the tract. The most medial and more dorsal parts of the medial septum were found to project within the medial fornix (Saunders & Aggleton, 2007), possibly suggesting greater termination in the posterior hippocampus. Fibers located in the middle of the coronal plane of the fornix, that is, in the intermediate part of the fornix, arose from more lateral cell populations in the medial septum and from the diagonal band of Broca. Interestingly, such a septo‐hippocampal topography has been reported in both the rat and the cat, where the most medial part of the medial septum and the ventromedial part of the diagonal band innervate the septal hippocampus, that is, the posterior hippocampus (Siegel, Edinger, & Ogami, 1974; Witter, 1986). In contrast, more lateral parts of the medial septum and the dorsomedial part of the diagonal band project upon the temporal (i.e., anterior) hippocampus.

Four white matter microstructural indices (FA, RD, *f*, and HMOA) were acquired for the two fornix subpopulations. Perhaps unsurprisingly, given the close anatomical proximity of the two subpopulations, all microstructural indices significantly correlated across the two fornix reconstructions. However, clear pathway differences arose when comparing each microstructural index for the anterior and posterior hippocampus, suggesting differences in the axonal organization of the two subpopulations. Anterior hippocampal/lateral fornix fibers exhibited significantly lower FA and lower HMOA as well as higher *f* and RD than posterior hippocampal/medial fornix fibers. The pattern of larger FA and HMOA together with lower RD suggests a more coherently aligned and more densely packed axon population in medial portions compared to lateral portions of the fornix. At the same time, the anterior hippocampus/lateral fornix fibers showed higher *f*, that is, a higher fraction of the signal attributable to tissue after the free water correction, than posterior/medial fibers. While the explanation for these differences remains unclear, contributing factors may include small differences in the angle of curvature and the extent to which the voxels include ventricular space. One further issue that could affect these comparisons concerns the way in which parahippocampal fibers pass through the posterior CA1 to join the medial fornix before crossing hemispheres in the dorsal hippocampal commissure (Demeter, Rosene, & Van Hoesen, 1985). In practice, the positioning of the “SEED” ROI around the body of the fornix should exclude much of this potential contribution as the large majority of fibers in this commissure cross caudal to this ROI (Demeter et al., 1985).

An implication of the tract reconstructions is that there are topographic distinctions within the medial–lateral dimension of the fornix that are likely to reflect corresponding differences in function along the longitudinal axis of the hippocampus. Of the two areas, the anterior hippocampus has been more linked to stress, anxiety, and emotional processing (Bannerman et al., 1999; Chase et al., 2015; O'Mara, 2005), whereas the posterior hippocampus is more typically linked to fine‐grain spatial processing (Poppenk et al., 2013; Strange et al., 2014). These differences do not, however, reflect a dichotomy, rather a gradation of change along the anterior–posterior axis (Collin et al., 2015; Poppenk et al., 2013; Strange et al., 2014). One interpretation is that the anterior hippocampus is important for forming large‐scale representations of the environment, whereas the posterior hippocampus provides more detailed representations (Poppenk et al., 2013; Strange et al., 2014). As a consequence, it has been proposed that relative differences along this axis include memory encoding, scene construction, and imagining events (anterior hippocampus), along with memory retrieval and spatial navigation (posterior hippocampus) (Poppenk et al., 2013; Zeidman & Maguire, 2016). The novel tractography protocol described here allows future investigations to examine the relationships between these different aspects of cognition, alongside the respective sets of white matter connections.

A previous study employing a similar methodology (Christiansen, Aggleton, et al., 2016) demonstrated that the fornix can also be divided between its precommissural and postcommissural fiber subdivisions (Poletti & Creswell, 1977). The precommissural fornix contains hippocampal connections with the basal forebrain, ventral striatum, and prefrontal cortex, whereas the postcommissural fornix connections reach the medial diencephalon and hypothalamus (Poletti & Creswell, 1977). Using diffusion imaging, the precommissural and postcommissural fibers were found to occupy different locations within the body of the fornix: the postcommissural fornix fibers were located dorsally, whereas the precommissural fibers were located ventrally (Christiansen, Aggleton, et al., 2016). Together with the present results, these findings suggest that there is a topography along the two planes of the fornix, that is, within both the dorsal–ventral and medial–lateral axes of the tract, with both topographies reflecting different sets of hippocampal connections. Consequently, hippocampal efferents to prefrontal cortex should first predominantly occupy the lateral fornix and then the dorsal fornix, going anteriorly along the body of the fornix.

## 5 Conclusions

This study demonstrated a clear separation of human fornical fibers depending on whether they were associated with the anterior or posterior hippocampus. Fibers associated with the anterior hippocampus were located laterally within the body of the fornix, whereas fibers associated with the posterior hippocampus were located medially. These findings pave the way for future work to determine whether these fornical subpopulations contribute to different cognitive functions and whether they are differentially affected by pathological conditions such as Mild Cognitive Impairment and Alzheimer's disease, both known to be associated with microstructural changes in the fornix (Fletcher et al., 2013; Metzler‐Baddeley, Hunt, et al., 2012; Sexton et al., 2010). The present findings offer, for example, a means to help determine whether there are differential rates of disruption in the white matter associated with the anterior and posterior hippocampus in these diseases.

## Conflict of interest

The authors have no conflict of interest to declare.
